# Novel mHealth App to Deliver Geriatric Assessment-Driven Interventions for Older Adults With Cancer: Pilot Feasibility and Usability Study

**DOI:** 10.2196/10296

**Published:** 2018-10-29

**Authors:** Kah Poh Loh, Erika Ramsdale, Eva Culakova, Jason H Mendler, Jane L Liesveld, Kristen M O'Dwyer, Colin McHugh, Maxence Gilles, Terri Lloyd, Molly Goodman, Heidi D Klepin, Karen M Mustian, Rebecca Schnall, Supriya G Mohile

**Affiliations:** 1 Division of Hematology/Oncology James P Wilmot Cancer Institute University of Rochester Medical Center Rochester, NY United States; 2 Department of Surgery (Cancer Control) James P Wilmot Cancer Institute University of Rochester Medical Center Rochester, NY United States; 3 Section on Hematology/Oncology Department of Internal Medicine Wake Forest Baptist Health Winston Salem, NC United States; 4 School of Nursing Coumbia University New York City, NY United States

**Keywords:** Mobile health application, geriatric assessment, older adults, cancer

## Abstract

**Background:**

Older patients with cancer are at an increased risk of adverse outcomes. A geriatric assessment (GA) is a compilation of reliable and validated tools to assess domains that are predictors of morbidity and mortality, and it can be used to guide interventions. However, the implementation of GA and GA-driven interventions is low due to resource and time limitations. GA-driven interventions delivered through a mobile app may support the complex needs of older patients with cancer and their caregivers.

**Objective:**

We aimed to evaluate the feasibility and usability of a novel app (TouchStream) and to identify barriers to its use. As an exploratory aim, we gathered preliminary data on symptom burden, health care utilization, and satisfaction.

**Methods:**

In a single-site pilot study, we included patients aged ≥65 years undergoing treatment for systemic cancer and their caregivers. TouchStream consists of a mobile app and a Web portal. Patients underwent a GA at baseline with the study team (on paper), and the results were used to guide interventions delivered through the app. A tablet preloaded with the app was provided for use at home for 4 weeks. Feasibility metrics included usability (system usability scale of >68 is considered above average), recruitment, retention (number of subjects consented who completed postintervention assessments), and percentage of days subjects used the app. For the last 8 patients, we assessed their symptom burden (severity and interference with 17-items scored from 0-10 where a higher score indicates worse symptoms) using a clinical symptom inventory, health care utilization from the electronic medical records, and satisfaction (6 items scored on a 5-point Likert Scale for both patients and caregivers where a higher score indicates higher satisfaction) using a modified satisfaction survey. Barriers to use were elicited through interviews.

**Results:**

A total of 18 patients (mean age 76.8, range 68-87) and 13 caregivers (mean age 69.8, range 38-81) completed the baseline assessment. Recruitment and retention rates were 67% and 80%, respectively. The mean SUS score was 74.0 for patients and 72.2 for caregivers. Mean percentage of days the TouchStream app was used was 78.7%. Mean symptom severity and interference scores were 1.6 and 2.8 at preintervention, and 0.9 and 1.5 at postintervention, respectively. There was a total of 27 clinic calls during the intervention period and 15 during the postintervention period (week 5-8). One patient was hospitalized during the intervention period (week 1-4) and two patients during the postintervention period (week 5-8). Mean satisfaction scores of patients and caregivers with the mobile app were 20.4 and 23.4, respectively. Barriers fell into 3 themes: general experience, design, and functionality.

**Conclusions:**

TouchStream is feasible and usable for older patients on cancer treatment and their caregivers. Future studies should evaluate the effects of the TouchStream on symptoms and health care utilization in a randomized fashion.

## Introduction

### Scope of the Problem

Older adults are more likely to receive cancer treatments with the increasing availability of these treatments possessing superior toxicity profiles and greater ease of administration. Compared to their younger counterparts, older adults have a higher prevalence of comorbidity, disability, and geriatric syndromes (eg, falls, functional decline, and delirium), putting them at an increased risk of treatment-related toxicities and adverse outcomes such as hospitalization and death [1-4]. A geriatric assessment (GA) is a compilation of reliable and validated tools to assess essential domains that are predictors of morbidity and mortality [5]. A GA can also guide interventions based on the impairments noted on the assessment, such as delivery of specific diet recommendations for nutritional deficits, referral to physical therapy and promotion of physical activity for physical performance problems, and assessment of medication adherence for patients with multiple health problems and are on many medications [6,7]. These evidence-based recommendations have been shown to improve outcomes such as nutritional status, frailty, and chemotherapy tolerance in older adults [8-11]. Nevertheless, implementation of GA-driven interventions is low in the oncology community [12,13].

Mobile health (mHealth) apps have the potential to monitor and deliver GA-driven interventions at home. Recent advances in information technology have allowed health care professionals to utilize apps in clinical practice [14,15]. In the cancer setting, mHealth apps have been designed for various uses which include providing education and support [16], monitoring symptoms and facilitating symptom reporting [17-20], monitoring medication adherence [21], promoting physical activity [22,23], and monitoring nutritional status and surgical care [24]. These apps collectively support a number of GA-driven interventions, but generally they are specialized to have a single focus (such as promoting physical activity), and only a limited number of them have been tailored specifically to older adults with cancer who have complex health care needs and for their caregivers who themselves frequently have health issues [25].

### Study Objectives

In this study, we utilized the TouchStream app [26]. It was designed by TouchStream Solutions (Rochester, New York, United States) with the goal of helping people live independently. Currently, it is being used primarily for patients with developmental disabilities. To evaluate if older adults with cancer can use the technology, we conducted this study to (1) evaluate the feasibility and usability of the TouchStream app to deliver GA-driven interventions and (2) identify barriers to use and issues with existing design and functionality. As an exploratory aim, we gathered preliminary data on symptom burden and health care utilization.

## Methods

### Study Design, Setting, and Sample

This prospective single-arm pilot study was conducted at the University of Rochester Medical Center (Rochester, New York, United States) from January to December 2017. Patients were recruited if they were aged ≥65 years, diagnosed with a solid tumor or hematologic malignancy, on systemic cancer treatment, able to understand and speak English, able to provide informed consent, and had a life expectancy of 6 months or greater. Patients were given the option to select a caregiver to participate in the study. A caregiver was defined as “a valued and trusted person in a patient’s life who is supportive in health care matters by providing valuable social support or direct assistive care.” The caregiver accompanies the patient to medical appointments, can listen and give thoughtful advice, and might be a family member, partner, friend, or professional caregiver. Caregivers had to be ≥21 years and able to understand spoken English and provide informed consent. Patients and caregivers were not required to have electronic devices with internet access to participate in the study as internet access was provided through the device using a wireless carrier.

### The TouchStream App

**Figure 1:**
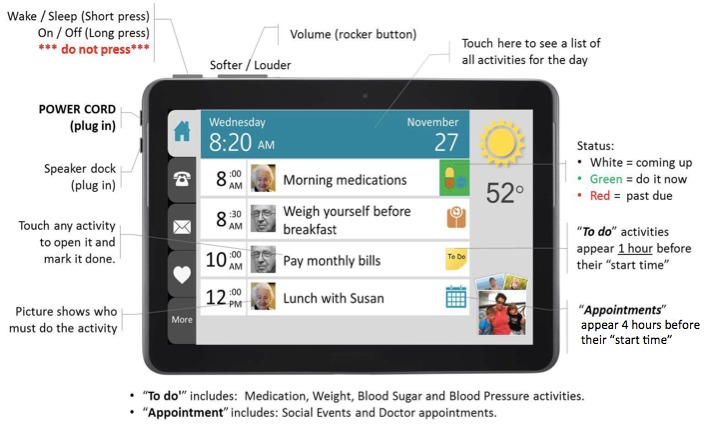
Tablet showing the interface of the mobile application.

**Figure 2:**
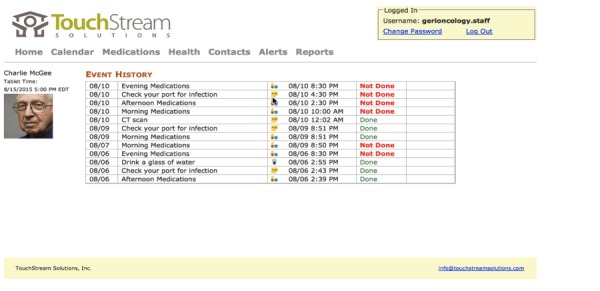
Interface of the Web portal

The TouchStream app was developed by TouchStream Solutions (Rochester, New York, United States). The app displays a list of activities entered from the Web portal and arranges them by the time of day (Figure 1). These activities include doctor appointments, medication reminders, monitoring, vital signs (eg, weight, blood pressure), surveys (eg, symptoms), contingency plans (eg, fever, constipation), and physical activity (in the form of daily steps). The study team entered activities tailored to the patient onto the Web portal before the start of the study based on the GA impairments (Table 1). At the appropriate date and time, the tablet speaks through a voice avatar reminding patients/caregivers to complete these activities. The app is connected to a Web portal (Figure 2). The Web portal is used to enter or remove activities, and it can be accessed using a desktop or laptop computer. The home page of the Web portal displays the patient’s information and a list of activities followed by the date and time and whether the tasks have been completed. This display allows the caregivers/patients and the study team to monitor for completion and compliance. All information entered on the Web portal is transferred to the app and vice versa. TouchStream stores data on both the tablet and TouchStream server. On the tablet, the data is encrypted, and the back-end server hosts a Microsoft Structured Query Language Server Database.

### Study Procedures

Once informed consent was obtained, all patients completed baseline questionnaires (on paper) that captured demographics and clinical information, their previous experience with electronic devices (ie, if they have access to any electronic devices and the total hours spent per week using these devices), and a symptom survey (see “Outcomes”). Clinical information was cross-checked with the electronic medical records for accuracy. Caregivers (available for 13 patients) also provided information on baseline demographics and their experience with electronic devices. All patients also underwent a baseline GA included measures of comorbidity (Older Americans Resources and Services (OARS) physical health section [27]), physical function (activities of daily living (ADL) [28], instrumental activities of daily living (IADL) [29], number of falls in the past year and Short Physical Performance Battery (SPPB) [30]), cognition (Blessed Orientation-Memory-Concentration (BOMC) [31] and Montreal Cognitive Assessment (MoCA) [32]), number of medications, social support (Medical Outcomes Study (MOS) Social Support Survey [33]), nutritional status (body mass index (BMI) and self-reported weight loss in the past 6 months) [34,35], and psychological status (Geriatric Depression Scale-15 (GDS-15) [36]). All measures were self-reported except for SPPB, BOMC, and MoCA that were performed by a study coordinator. The GA was performed to uncover baseline impairments as well as to guide interventions or activities delivered through the TouchStream app (Table 1). The list of GA-driven interventions was based on a prior study and represented a consensus from geriatric oncology experts on how GA can guide nononcologic interventions [6]. Based on this, we selected interventions that can be delivered through the mobile app and adapted them for our study.

After the baseline assessment, the study team entered activities tailored to the patient onto the Web portal. Patients and caregivers were provided with a touchscreen tablet connected to a data plan for internet access and preloaded with the TouchStream app in addition to chargers and instruction manuals for use at home. Patients were initially also provided with a speaker and a cable that connects the speaker to the tablet, but these were removed during the study period for simplicity. A stylus was also provided for use if patients had difficulty with the touchscreen. The study team provided a brief tutorial on how to use the TouchStream app and Web portal to both the patients and caregivers. Patients were then asked to use the app for the following 4 weeks, and caregivers were asked to assist the patients if needed. Patients and caregivers were also given the option to access the Web portal to enter additional activities if they wished to during the study period.

They were asked to place the tablet at a place of choice (eg, kitchen, living room, bedroom, or study room). Any activities delivered through the app were for the patients and primarily informational for the caregivers. Patients were encouraged to bring the tablet with them when they left the house. The study team accessed the Web portal at least once weekly and on an as-needed basis to enter new activities and monitor existing activities. If any concerns were noted (eg, patient-reported pain for several days in a row), the study team communicated these concerns to the primary oncology team. During this time, the study team and TouchStream Solutions were available to both the patients and caregivers for questions and technical assistance.

**Table 1:** Geriatric assessment domains, tools, and interventions.

Domain	Tool	Score signifying impairment	Interventions/activities
Comorbidity	OARS^a^ physical health section	≥5 illnesses that affected them by a “great deal” ≥3 illnesses that affected them by “somewhat,” or vision/hearing rated as “fair, poor, or totally blind/deaf”	Access to a list of the patient’s medical conditions
Physical function	ADL^b^ IADL^c^ Fall history SPPB^d^	Any ADL or IADL impairment Fall(s) within the past year ≤9 on SPPB	Handouts on energy conservation via the tablet with reminders Exercise and fall counseling provided through the tablet Daily steps monitoring and reminders for increasing physical activity^e^
Cognition	BOMC^f^ MoCA^g^	>4 on BOMC <26 on MoCA	Reminders for medications and appointments
Polypharmacy	No. of total medications	≥5 medications	Medication (scheduled and as needed) reminders and monitoring Provide instructions including dosages, frequencies, and indications for all medications to patients and caregivers Automated reminders to caregivers if patients missed their medications
Social support	MOS^h^ medical social support	Any deficit noted	Easy access to caregiver and health care teams’ contact information
Nutrition	BMI^i^	BMI of <21 >5% weight loss in the last six months	Provide recommendations and reminders for hydration Nutritional handouts
Psychological health	GDS-15^j^	≥5 on GDS-15	Monitoring of distress and mood
All patients	—^k^	—	Cancer treatment information including regimen and dose Contingency plans related to their treatment (eg, constipation, diarrhea, and fever) Symptom monitoring

^a^OARS: Older Americans Resources and Services.

^b^ADL: activities of daily living.

^c^IADL: instrumental activities of daily living.

^d^SPPB: Short Physical Performance Battery.

^e^Patients were encouraged to enter the number of steps during the study if they have a step counter. If they did not have a step counter, they were asked to enter the approximate number of steps based on distance walked.

^f^BOMC: Blessed Orientation-Memory-Concentration.

^g^MoCA: Montreal Cognitive Assessment.

^h^MOS: Medical Outcomes Study.

^i^BMI: body mass index.

^j^GDS-15: Geriatric Depression Scale-15.

^k^Not applicable.

**The system usability scale questionnaire.**

1. I think I would like to use this system frequently.
2. I found the system unnecessarily complex.
3. I found the system was easy to use.
4. I think I would need the support of technical person to be able to use this system.
5. I found the various functions in this system were well integrated.
6. I thought there was too much inconsistency in this system.
7. I would imagine that most people would learn to use this system very quickly.
8. I found the system very cumbersome to use.
9. I felt very confident using the system.
10. I needed to learn a lot of things before I could get going with this system.

At the end of the study period, patients and caregivers returned to meet with the study team for a semistructured interview (approximately 30 minutes to an hour) to obtain feedback about the app, including functionality, design, and barriers to use. The interviews were audio-recorded. Both patients and caregivers also completed postintervention assessments that included usability and symptom surveys.

### Outcomes

The primary outcome was usability assessed by the system usability scale (SUS). The SUS is a standardized questionnaire commonly used to assess participants’ perceptions of usability of an electronic system or device [37,38]. The scale consists of 10 items, and each item is rated on a 5-point Likert scale (Textbox 1). A score higher than 68 is considered above average in the evaluation of mHealth apps [37,38].

Other feasibility metrics included recruitment rate (no. of subjects recruited divided by the no. of patients approached), retention rates (no. of subjects consented who completed postintervention assessments), the percentage of days the tablet was turned on, and percentage of days subjects used the app.

The scores for each question are converted to a new number using the following formula: odd-numbered questions are calculated as the scale position minus 1, and even-numbered questions are calculated as 5 minus the scale position. The scores are added together and multiplied by 2.5 to get the final score, with a range of 0 to 100.

Additionally, as prespecified in the protocol, for the last 8 patients enrolled in the study, we gathered data on patients’ and caregivers’ satisfaction as well as patients’ symptom burden and health care utilization. The modified satisfaction survey consisted of 6 items, and patients and their caregivers (if available) rated each question on a 5-point Likert scale, with a total score of 30 and a higher score indicating greater satisfaction [39]. Symptom burden was assessed using a clinical symptom inventory [40]. Patients were asked to rate the severity of 11 symptoms (eg, pain, nausea, disturbed sleep) at its worst in the past week from 0 (not present) to 10 (as bad as you can imagine). They were also asked to rate how the symptoms had interfered with their lifestyle in 6 domains: (1) general activity, (2) mood, (3) work, (4) relations with other people, (5) walking, and (6) enjoyment of life. Health care utilization during the study period (week 1 to 4) and postintervention period (week 5-8) was obtained from the electronic medical records by the study team. Utilization metrics captured included numbers and types of clinic calls, number of missed appointments, and hospitalizations.

### Analyses

Descriptive analyses (count, mean, SD, range, and percentage as appropriate) were used to describe the study sample demographics and GA findings, feasibility metrics, and outcomes. Qualitative interviews were transcribed. Two coders reviewed and coded these transcripts using conventional content analysis [41], focusing on users’ experiences and their feedback on the design and functionality of the app including ease and barriers of use. Any discrepancies were resolved through discussion.

## Results

### Baseline Characteristics

From January to December 2017, 30 patients were approached and 20 patients and 14 caregivers consented to the study (recruitment rate 66.7%). Two patients and 1 caregiver did not complete baseline assessment (1 patients did not provide any reason while another patient had “too much going on”), resulting in a total sample of 18 patients and 13 caregivers. Table 2 shows the baseline characteristics for patients and caregivers. Mean ages of the patients and caregivers were 76.8 (SD 5.4, range 68-87) and 69.8 (SD 13.5, range 38-81), respectively. The majority of patients were male (15/18, 83%) while most caregivers were female (12/13, 92%). They were predominantly white (patients: 16/18, 89%; caregivers: 11/13, 85%) and married (patients: 13/18, 72%; caregivers: 11/13, 85%). More than half of the patients (12/18, 67%) and caregivers (7/13, 54%) completed college or university education. Many patients (15/18, 83%) had at least one caregiver at home, most of whom were their spouses or significant others (14/18, 78%).

Concerning the type of cancer, 78% (14/18) of the patients had hematologic malignancies. Most of these patients were on hypomethylating agents. The mean number of GA impairments was 4.6 (SD 1.9, range 1-7), 17% (3/18) had up to two impairments, 39% (7/18) had three to five impairments, and 44% (8/18) had six or more impairments. Table 2 shows impairments in the various domains.

### Experience With Electronic Devices

Most patients and caregivers had access to electronic devices, with desktop and laptop being the common (Table 2). Among the patients, 8 of 18 (44%) had access to a mobile phone and 3 of 18 (17%) had access to a tablet or iPad. Among the caregivers, 8 of 13 (62%) had access to a mobile phone and 4 of 13 (31%) had access to a tablet or iPad. Over half (10/18, 56%) of the patients and (9/13, 69%) of the caregivers spent more than five hours a week on their own electronic devices.

### Retention Rate, Usability, Feasibility, and Satisfaction

During the study period 1 of the 18 (6%) patients left as she was no longer interested in the study. Another (1/16, 6%) patient and (1/13, 8%) caregiver had hearing difficulties and did not want to continue being involved in the study. One of the 13 (8%) caregivers did not complete the postintervention assessment due to the inability to come to the study visit. The retention rates for patients and caregivers were 89% (16/18) and 85% (11/13), respectively.

The mean SUS score was 74.0 (SD 14.5, range 22.5-100.0) for patients and 72.2 (SD 22.2, range 45.0-92.5) for caregivers. Mean percentage of days the tablet was turned on was 88.7% (SD 14.1, range 47-100), and the mean percentage of days the mobile app was used was 78.7% (SD 18.6, range 37-100). Ninety-four percent used the app for more than 50.0% of the study days.

Mean satisfaction scores of patients (n=8) and caregivers (n=5) with the TouchStream app were 20.4 (SD 6.6) and 23.4 (SD 8.1), respectively (Table 3).

### Symptom Burden and Health Care Utilization

Mean symptom severity score was 1.6 (SD 1.0, range 0.2-4.6) at preintervention and 0.9 (SD 0.6, range 0-3.5) at postintervention. Mean symptom interference score was 2.8 (SD 1.2, range 0-4.2) at preintervention and 1.5 (SD 1.5, range 0-5.3) at postintervention.

Among the 8/18 (44%) patients for whom health care utilization was assessed, there was an average of 3.4 (total=27, SD 3.1, range 1-12) clinic calls during the intervention period (week 1-4) and 1.9 clinic calls (total=15, SD 1.6, range 0-5) during the postintervention period (week 5-8). The majority of phone calls were related to appointments, followed by symptom reporting, and medication advice. One of 18 (6%) patients was hospitalized during the intervention period (week 1-4) and 2/18 (11%) patients during the postintervention period (week 5-8). Two of 18 (11%) patients had missed appointments due to factors unrelated to cancer or its treatment during the intervention period, and none during the postintervention period.

### Semistructured Interviews

#### Theme 1: General Experience

Many patients (10/16, 63%) and caregivers (8/11, 73%) appreciated and enjoyed the experience, and saw the value of the TouchStream app. Four patients (4/16, 25%) commented that the app would be good for someone living alone and 1 patient (6%) suggested that it would be helpful for home care nurses to help with home monitoring. It could also be useful patients who have memory impairment. One patient thought that the app helped him connect to the team more easily.

It is an exceptionally good idea to have a companion on the team. You extended the team back into my house, and that was great.

Patient #13, male

**Table 2:** Baseline demographic and clinical characteristics, geriatric assessment impairment, and patient/caregiver experience with electronic devices.

Variables				Patient (n=18), n (%)		Caregiver (n=13), n (%)
**Demographic and clinical characteristics**						
	Age in years, mean (SD, range)			76.8 (5.4, 68-87)		69.8 (13.5, 33-81)
	**Gender**					
			Male		15 (83)	1 (8)
			Female		3 (17)	12 (92)
	**Race**					
			White		16 (89)	11 (85)
			Other		2 (11)	2 (15)
	**Marital status**					
			Married		13 (72)	11 (85)
			Long-term committed significant other		2 (11)	1 (8)
			Widow		3 (17)	0
			Divorce		0	1 (8)
	**Education level**					
			Postgraduate		5 (28)	4 (31)
			College/university		7 (39)	3 (23)
			Some college/university		4 (22)	3 (23)
			High school/GED^a^ or lower		2 (11)	3 (23)
	**Caregiver(s) at home^b^**					
			Spouse/significant other		14 (78)	—
			Child/children		1 (6)	—
			Grandchild/grandchildren		2 (11)	—
			None		3 (17)	—
	**Caregiver(s) not living at home^b^**					
			Child/children		5 (28)	—
			Other relative(s)		3 (17)	—
			Friend(s)		2 (11)	—
			None		9 (50)	—
	**Relationship with the patient**					
			Spouse/significant other		—	11 (85)
			Child/children		—	1 (8)
			Other relative		—	1 (8)
		**Cancer subtype**				
			Leukemia		8 (44)	—
			Myelodysplastic syndrome		4 (22)	—
			Lymphoma		2 (11)	—
			Solid tumors (esophagus, prostate, and lung)		4 (22)	—
	**Treatment**					
			Hypomethylating agents		11 (61)	—
			FOLFOX^c^-based		2 (11)	—
			Rituximab-based		2 (11)	—
			Other		3 (17)	—
**Geriatric assessment impairment**						
	Comorbidity (OARS^d^)			12 (67)		—
	ADL^e^ (≥1 impairment)				0	—
	IADL^f^ (≥1 impairment)				9 (50)	—
	Falls (≥1 in the past year)				5 (28)	—
	Objective physical function (SPPB^g^; ≤9)				14 (78)	—
	Cognition (BOMC^h^ or MoCA^i^)			10 (56)		—
	**Polypharmacy**					
			≥5 medications		16 (89)	—
			Nutrition (% weight loss or BMI^j^)		7 (39)	—
	Depression (GDS-15^k^; ≥5)				5 (28)	—
	Social support (MOS^l^)			4 (22)		—
**Experience with electronic devices**						
	**Access to electronic devices^b^**					
			Desktop		9 (50)	10 (77)
			Laptop		11 (61)	5 (39)
			Mobile phone		8 (44)	8 (62)
			Tablet/iPad		3 (17)	4 (31)
	**Total hours spent/week on own device(s)**					
			0-5		8 (44)	4 (31)
			6-10		4 (22)	3 (23)
			11-15		3 (17)	4 (31)
			16-20		1 (6)	1 (8)
			>20		2 (11)	1 (8)

^a^GED: General Equivalency Development.

^b^Total percentage does not equal to 100%.

^c^FOLFOX: folinic acid, fluorouracil, and oxaliplatin.

^d^OARS: Older Americans Resources and Services.

^e^ADL: activities of daily living.

^f^IADL: instrumental activities of daily living.

^g^SPPB: Short Physical Performance Battery.

^h^BOMC: Blessed Orientation-Memory-Concentration.

^i^MoCA: Montreal Cognitive Assessment.

^j^BMI: body mass index.

^k^GDS-15: Geriatric Depression Scale-15.

^l^MOS: Medical Outcomes Study.

**Table 3:** Patients’ and caregivers’ satisfaction scores with the TouchStream mobile app.

Statements and possible score (range 1-5)		Mean, (SD, range)
**Patients (n=8)**		
	Overall satisfaction using the app	3.8 (1.2, 2-5)
	The app helped improve the care coordination for my cancer	3.6 (1.3, 1-5)
	The app helped with my appointments	3.0 (1.5, 1-5)
	The app helped with my medications	3.6 (1.2, 1-5)
	The app helped me with the management of side effects from cancer treatments	3.0 (1.4, 1-5)
	I would recommend TouchStream to my family and friends	3.4 (1.2, 1-5)
	Total (possible range 5-30)	20.4 (6.6, 7-30)
**Caregivers (n=5)**		
	Overall satisfaction using the app	4.2 (1.8, 2-5)
	The app helped improve the care coordination for his/her cancer	3.6 (1.7, 1-5)
	The app helped with his/her appointments	3.5 (1.9, 1-5)
	The app helped with his/her medications	4.6 (0.9, 1-5)
	The app helped him/her with the management of side effects from cancer treatments	3.4 (1.7, 1-5)
	I would recommend TouchStream to my family and friends	3.8 (1.8, 1-5)
	Total (possible range 5-30)	23.4 (8.2, 10-30)

Three patients (3/16, 19%) already had involved caregivers who provided the same services as the tablet, although the caregivers themselves appreciated the mobile app. One patient (6%) felt that his primary oncology team was already very responsive. Another patient (6%) also commented that the app might be challenging for someone who is computer illiterate. Also, the app was challenging for patients who were immobile, as the tablet was set up in one place at home. Those who were still working or spending most of their time outdoors were not able to attend to the activities unless they brought the tablet with them, and they preferred the idea of a mobile phone-based app. Three patients (3/16, 19%) suggested integration with wearable technologies (eg, smartwatch).

I like something a little more mobile, like my iPhone, like an app on my iPhone. This (the tablet) is big but I am always near it to use it and I did answer the questions, maybe not right at the time but near it.

Patient #5, male

#### Theme 2: Design

The majority of patients/caregivers did not encounter any major barriers with the design and commented that it was easy to use (1 patient was technologically illiterate and was not able to use it). The brightness and the font and screen sizes were appropriate for this age group. Only 1 patient utilized the instruction manuals, and most commented that they only needed a few days to get used to the app. After that, they were able to use it regularly.

Three patients (3/18, 19%) had difficulty with the touchscreen and 2 of these patients were able to use it with a stylus. One patient did not like touchscreen devices and preferred to interact with a device through a physical button. One patient did not like the monotone voice from the tablet and wanted additional selections, while one caregiver (1/11, 9%) preferred the monotone voice as it could not be confused with someone in the house. One patient preferred a smaller screen size while another patient preferred one that was bigger.

I used the stick [stylus], I tried my finger and I realized it wouldn’t always respond. I did eye screening for little kids [for my job] and you have to punch in all these things, and I do it with my finger on a touchscreen so I am used to doing that but this screen didn’t seem to respond to my finger.

Patient #20, male

#### Theme 3: Functionality

The various functions including appointment, medication, and nutritional reminders were helpful to some patients and caregivers. The medication reminders (scheduled and as needed) encouraged patients/caregivers to think about the indications for the medications and whether these medications were necessary. The daily step reminders made patients conscious of their physical activity. However, these reminders were not sufficient enough to promote physical activity in and of themselves. One patient suggested that exercise recommendations from his oncologist would be helpful in combination with the app. Contingency plans related to their treatment (eg, constipation, diarrhea, fever) were helpful for patients who were receiving their first few cycles of treatment but not for those who had been receiving treatment for a longer period. The list of activities was beneficial for them to keep track of things. The reminders when conveyed through the voice avatar or listed on the tablet also generated conversation between patients and caregivers and other family members and friends who were not involved in the care of the patients.

It may be helpful for the caregiver to know what you have done and when. They can check the tablet because you (the patient) may not want to talk about it or may not have remembered.

Caregiver #13, female

Three patients (3/16, 19%) already had a system to keep track of activities such as appointments and medications, and therefore did not find the reminders helpful. The symptom survey was overall not very helpful to patients and caregivers, as no feedback was provided on the tablet after they filled out the survey. However, 1 patient did recommend optional daily symptom surveys, recognizing that symptoms can fluctuate and may be missed by more infrequent surveys. Patients also preferred the ability to enter open-ended answers in the surveys. They were unable to ignore the reminders or erase their answers on the surveys or tasks once they had been filled out. One patient also did not want to be continuously reminded that she was sick.

It would be nice to have some daily jokes or something educational... to just always be reminded that you are sick, you need to do this, you need to do that; you know we have many stuff going on.

Patient #4, female

Only 1 patient (1/16, 6%) and 2 caregivers (2/11, 18%) accessed the Web portal to add or remove activities. Patients and caregivers thought that the health care team should be responsible for entering these activities, and would prefer that the TouchStream system be integrated with their electronic medical records.

## Discussion

### Principal Findings

In this pilot study, we demonstrated that the TouchStream mobile app is a usable and feasible platform with which to deliver GA–driven interventions for older adults with cancer and their caregivers. Many older patients and caregivers own electronic devices, and they are open to participating in studies testing a mobile technology device. We also showed that it is feasible to monitor symptoms and health care utilization in this vulnerable population as part of a clinical study.

Mobile technologies are increasingly used for health purposes even among older adults who have demonstrated a lower uptake of technologies compared to younger adults [42]. These technologies have the potential to assist in care coordination activities for older adults with cancer. However, most mobile apps are not designed specifically for this population who have complex health care needs. In addition, caregivers who are involved in the care of older adults with cancer are rarely included in studies evaluating mobile technologies. In this study, we gathered input and identified barriers to the use of a mobile app from the patients’ and caregivers’ perspectives. These barriers are currently being used to refine and improve the TouchStream system. Also, we propose a set of recommendations for future studies that aim to evaluate apps for older adults with cancer focusing on general issues as well as the design and functionality of the app (Table 4).

### Additions to the Literature

Using the Delphi technique, Mohile and colleagues [6] previously developed an algorithm to help guide nononcologic interventions based on the GA. These interventions were converted to activities and were tailored for each patient based on their GA findings. Multiple mHealth apps intended to enhance and promote self-management have been designed for patients with chronic illnesses including cancer, though most of them have generic functions and are not tailored to individual patients [43,44]. Our approach is novel and innovative as we tailored the interventions to the patients.

**Table 4:** Recommendations for future studies utilizing a mobile technology device.

Domain	Recommendations
General	Coordinate study visits with clinic or treatment appointments Simplify instructions and accompanying accessories (eg, a built-in speaker with a range of volume, one cable, and video demonstration) Ensure internet access is reliable Engage caregivers and treatment team including homecare nurses if possible
Design	Provide stylus for touchscreen devices or utilize devices with buttons or a remote Provide a list of voice options Provide the options for smartphone and tablet-based app (for both patients and caregivers) Provide a mobile device with varying screen sizes Ensure the screen color, font size, and brightness are appropriate for the study population
Functionality	Tailor the interventions and activities to each individual If symptom reporting is incorporated, ensure that feedback is provided after symptoms have been reported When surveys are administered, allow users to enter open-ended answers and to change or erase answers Interface the app with electronic health records (to ensure consistency of information) Provide a digital activity tracker when exercise intervention is recommended with the ability to sync exercise data from the tracker to the app automatically Provide an option for users to enter activities through the mobile application in addition to the Web portal Set an appropriate frequency for reminders (to ensure compliance but not to overburden users) Incorporate nonmedical functions such as social and educational activities and daily jokes or words

In our patient and caregiver interviews, many expressed appreciation and valued the experience. Our goal is to optimize this platform using their feedback and suggestions to allow incorporation of other GA-driven interventions that can be delivered through the mobile app (eg, cognitive rehabilitation for cognitive impairment, cognitive behavioral therapy for psychological impairment, MedicAlert bracelet that interacts with the app).

### Study Limitations

Our study has several limitations. First, this is a single center study with a small sample size and predominantly male patients and female caregivers. During accrual, a higher number of female patients did not want to participate. A common reason provided was that they already had a system in place to help with managing their care. Our sample was also highly educated. All of these may limit generalizability to a larger population of patients with cancer. Second, we did not statistically compare the changes in outcomes due to the heterogeneity of our patient population and small sample size. We acknowledge that symptoms and health care utilization are highly dependent on the type of cancer, the stage of the disease, and the treatment(s) administered, and our sample had varying durations of treatment ranging from one month to several years. Third, patients and caregivers were provided with the tablet for approximately 4 weeks with a relatively short follow-up. For future studies, we plan to extend both the intervention and follow-up periods.

### Conclusion

In conclusion, we demonstrated that the TouchStream mobile app is feasible and usable for older patients undergoing cancer treatment and for their caregivers. Older patients and their caregivers value the experience of using an app in the management of their care, but the design and functionality of mobile technologies need to be adapted and tailored to their needs. Future studies should evaluate the effects of the TouchStream app on symptoms and health care utilization in a randomized fashion.

## Abbreviations

ADL: activities of daily living
BMI: body mass index
BOMC: Blessed Orientation-Memory-Concentration
FOLFOX: folinic acid, fluorouracil, and oxaliplatin
GA: geriatric assessment
GDS-15: Geriatric Depression Scale-15
GED: General Equivalency Development
IADL: instrumental activities of daily living
mHealth: mobile health
MoCA: Montreal Cognitive Assessment
MOS: Medical Outcomes Study
OARS: Older Americans Resources and Services
SPPB: Short Physical Performance Battery
SUS: system usability scale
